# Barriers and enablers for participation in healthy lifestyle programs by adolescents who are overweight: a qualitative study of the opinions of adolescents, their parents and community stakeholders

**DOI:** 10.1186/1471-2431-14-53

**Published:** 2014-02-19

**Authors:** Kyla L Smith, Leon M Straker, Alexandra McManus, Ashley A Fenner

**Affiliations:** 1 School of Physiotherapy and Exercise Science, Curtin University, Perth, Australia; 2 Curtin Health Innovation Research Institute, Curtin University, Perth, Australia; 3 School of Psychology and Speech Pathology, Curtin University, Perth, Australia

**Keywords:** Adolescent, Obesity, Intervention, Qualitative research

## Abstract

**Background:**

Overweight or obesity during adolescence affects almost 25% of Australian youth, yet limited research exists regarding recruitment and engagement of adolescents in weight-management or healthy lifestyle interventions, or best-practice for encouraging long-term healthy behaviour change. A sound understanding of community perceptions, including views from adolescents, parents and community stakeholders, regarding barriers and enablers to entering and engaging meaningfully in an intervention is critical to improve the design of such programs.

**Methods:**

This paper reports findings from focus groups and semi-structured interviews conducted with adolescents (n?=?44), parents (n?=?12) and community stakeholders (n?=?39) in Western Australia. Three major topics were discussed to inform the design of more feasible and effective interventions: recruitment, retention in the program and maintenance of healthy change. Data were analysed using content and thematic analyses.

**Results:**

Data were categorised into barriers and enablers across the three main topics. For recruitment, identified barriers included: the stigma associated with overweight, difficulty defining overweight, a lack of current health services and broader social barriers. The enablers for recruitment included: strategic marketing, a positive approach and subsidising program costs. For retention, identified barriers included: location, timing, high level of commitment needed and social barriers. Enablers for retention included: making it fun and enjoyable for adolescents, involving the family, having an on-line component, recruiting good staff and making it easy for parents to attend. For maintenance, identified barriers included: the high degree of difficulty in sustaining change and limited services to support change. Enablers for maintenance included: on-going follow up, focusing on positive change, utilisation of electronic media and transition back to community services.

**Conclusions:**

This study highlights significant barriers for adolescents and parents to overcome to engage meaningfully with weight-management or healthy lifestyle programs. A number of enablers were identified to promote ongoing involvement with an intervention. This insight into specific contextual opinions from the local community can be used to inform the delivery of healthy lifestyle programs for overweight adolescents, with a focus on maximising acceptability and feasibility.

## Background

It is estimated that one quarter of adolescents in Australia are overweight or obese [1,2] with adolescence recognised as a prime time for significant progression of obesity [3]. It has been suggested that changes to environmental and societal factors such as a decrease in physical activity, an increase in sedentary behaviour and the availability of low cost high fat, high energy food have contributed to these high rates of overweight and obesity [4]. Obesity during adolescence is related to adverse health outcomes including hypertension, orthopaedic complications, sleep apnoea, increased risk of Type II diabetes [4,5], poor self-esteem and depression [6,7]. Adolescence is therefore a critical point for the implementation of effective prevention and management initiatives. The most recent Cochrane Review suggests that promoting a healthy lifestyle through a family-based program with a focus on improving diet and activity behaviours is the most effective way to manage overweight and obesity at this age [8].

There have been a small number of high-quality long-term trials to evaluate family-based obesity management in adolescence, with most reporting limited success [9-16]. The literature suggests a trend of modest anthropometric improvements immediately post-intervention, but an absence of evidence to suggest sustained long-term changes [17-19]. Further, minimal information on behavioural changes by participants has been reported and with most research reporting on outcomes rather than the process measures such as methods used to attract participants or program delivery [20], there is limited evidence about how to achieve such changes. Thus how to most effectively and appropriately change the health trajectory for overweight adolescents remains unanswered. From efficacy, health services planning and ethical points of view, there is much to be gained from a more extensive evidence base in this area [8]*.*

Nguyen et al. [21] reported articles in school newsletters and community newspapers as the most effective means of recruiting overweight adolescents, however, stated that these two strategies alone would be insufficient to yield enough participants. Once adolescents or parents have learnt of a treatment option, there is even less information about the processes involved in the initiation of care. This is of concern as noted by a Canadian research team that suggest around 50% of referrals to weight management programs do not attend their first appointment [22].

For those who do seek treatment, there is limited evidence regarding prevention of attrition and ways of keeping adolescents engaged. A review of the literature relating to attrition from paediatric weight management programs suggest that between 27% and 73% of participants drop out of interventions [23]. It appears that patients with greater health risks were more likely to drop out of treatment, as were ‘vulnerable’ families (e.g. minority groups, single parent families) although this was not conclusive [23]. Although all participants were thought to face some barriers to participation, it seemed that program non-completers perceived more barriers to participation than those who completed treatment [24]. Other family factors that may impact on attrition included unmet expectations, too much information to learn, cost, and scheduling conflicts [23,24]. There has been some success in the United States (US) where the cost of participation has been offset by government funded health schemes however this retention strategy is costly and not universally accepted due to differing health care system protocols [22]. In-depth interviews with paediatric clinicians suggest that while most health professionals recognise attrition as a major issue, there is no consensus about how to manage it [25]. Whilst some ideas for keeping families engaged in programs have been proposed, such as building positive relationships with program staff, meeting or managing parent and child expectations and building child confidence [24,25], there is insufficient detailed information on the opinions of adolescents and their families on what is important to maintain engagement in a program.

There is also a gap in the literature about how to encourage maintenance of healthy behaviour change post-program. In adults, clinical trials focussing on lifestyle components (activity and dietary behaviours) have demonstrated long term successes with maintained reduction in weight [26,27]. The literature tends to have a greater focus on initial weight loss than ongoing weight maintenance and reporting of longer term outcomes is limited by high drop-out rates and a lack of intent-to-treat data for subjects who may not have been as successful with weight loss [26,28,29]. There have been very few long term studies in youth and of those, the focus has been on 6–12 year olds [30]. Indications from the literature suggest that there is better maintenance of weight loss in youth than observed in adults which supports the importance of early intervention [29]. Behaviours like reduced television viewing and regular consumption of breakfast have been linked to weight maintenance, as has maintaining meaningful contact with clinicians involved in treatment [27,31]. There is still limited evidence on how to best encourage maintenance of healthy lifestyle changes in adolescents.

Qualitative research exploring the barriers and enablers to complex health interventions can provide a better evidence base to inform practitioners and policy makers about what is needed to achieve successful interventions [8,32]. A recent report [33] identified a number of strategies for recruitment and retention to general community based healthy lifestyle programs. These included encouraging positive word of mouth, fostering strong links with community groups and distributing printed materials in a range of ways including within school newsletters, targeted mail-outs and posting in community venues. However, the report also identified that different strategies may be needed for different population groups. The opinions of local community members, past and potential weight management or healthy lifestyle program participants and interested stakeholders, are thus likely to be useful in developing an understanding of what might and might not work for interventions targeting adolescents who are overweight.

In this study, focus groups and semi-structured interviews were conducted with adolescents, parents and community stakeholders to provide rich insights into the experiences and perceptions of these groups. The aim of the study was to identify key individual, family and community enablers and barriers to the implementation of a multi-disciplinary family-centred intervention for overweight adolescents to be delivered in a community setting; particularly in relation to recruitment of families, retention of families and maintenance of healthy changes. The insight into specific contextual opinions from the local community can be used to inform the delivery of healthy lifestyle programs for overweight adolescents, with a focus on maximising acceptability and feasibility.

## Methods

### Participants

Participants for the current study were recruited from families who had completed the Curtin University Activity, Food and Attitudes Program (CAFAP), potential CAFAP participant families and community stakeholders. CAFAP is an 8-week healthy lifestyle program for adolescents and their parents and was run as a pilot program during school terms in 2009 and 2010 in Perth, Western Australia [34]. The research team adapted a successful adolescent obesity tertiary hospital program (Princess Margaret Hospital ‘Fitmatters’ program) and delivered it within a university community context. The program was run by a dietitian, physiotherapist and psychologist and focussed on development of healthy lifestyle behaviours. The adaption was based on the available evidence [8] and informed by the research group’s professional experience. The participants in this pilot program were female (n?=?22) and male (n?=?8), obese (BMI percentile mean 96) and aged between 12 and 16 years.

In this study, past participant inclusion criteria was an adolescent aged 12–16 years with a previous attendance of at least 6 CAFAP sessions and a BMI-for-age greater than the 85th percentile [3] when they entered the program, or the parent of such an adolescent. Potential participant inclusion criteria was an adolescent aged 12–16 years, or the parent of an adolescents aged 12–16 residing in Western Australia. Stakeholder inclusion criteria included adults working with youth, childhood obesity or related community services.

All families who had completed CAFAP (n?=?30) were invited to participate and we aimed to recruit 7 adolescents and 7 parents. Adolescents and their parents/carers were initially offered a written invitation to attend focus groups, and follow-up emails and telephone calls were used to maximise attendance, along with a voucher incentive. Participants were given the option of completing a survey electronically if unable to attend a focus group due to timing or transport issues. Separate focus groups for adolescents and parents were planned to encourage open discussion. Past participants were invited to participate to provide a range of opinions based on their experience of a healthy lifestyle program.

Potential participants who had not been influenced by previous involvement in a healthy lifestyle program were invited to participate to provide a range of opinions based on their naïve perceptions of such a program. Recruitment was by referral from General Practitioners, school nurses, and other health professionals, as well as advertisement through community newspapers, school newsletters and radio. As for past participants, separate groups were planned for adolescents and parents. We aimed to recruit 24 adolescents and 24 parents.

Stakeholders with an interest in youth, childhood obesity or community services were invited to participate in a one-off interview. Health professionals in Western Australia and researchers from across Australia, as well as community organisation representatives and policy makers from two metropolitan areas and a regional town were approached based on their experience or interest in overweight and obesity during adolescence. The metropolitan areas chosen included areas of low socio-economic status and were the likely sites for a future intervention, thus providing appropriate local context to inform future delivery. Stakeholders were chosen to reflect a range of diverse views from professionals with an interest or experience in adolescent obesity. We aimed to initially interview 12 community stakeholders and based on their recommendations would interview others identified as having useful experience or insight.

Ethical approval for this research has been obtained from Curtin University Human Research Ethics Committee (HR105/2011). Written informed consent was provided by all participants. This research was conducted in accordance with the Helsinki Declaration of Human Rights.

#### Focus group and interview content

The theoretical foundation for this study was based on the Ecological Systems Theory (EST) proposed by Brofenbrenner [35], which suggests a complex model of interacting factors impacting human development. The application of EST by Davison and Birch [36] describes an interplay of risk factors in the development of childhood overweight occurring at a number of ecological levels. In relation to our study, EST offers a framework to consider the context of an adolescent’s life in the realms of familial, school, community and greater social environments. Participants for this study were chosen to reflect each level and thus included adolescents (individual), parents (familial) and stakeholders (from school, community and social environments). Questions were tailored to each audience to explore three priority areas relating to overweight adolescent healthy lifestyle programs, specifically: recruitment, retention and long-term maintenance. The main questions were developed with input from the multidisciplinary research team, with a number of sub-questions to fully explore barriers and enablers to effective program delivery. Proposed questions were further reviewed by a panel (including health promotion, physical activity, nutrition, psychology, social work, exercise physiology and behaviour expertise). Schedules were trialled and modified accordingly. Copies of the different schedules are provided in Additional files 1 and 2. The schedules were used by facilitators to guide discussion ensuring specific topics were covered, whilst allowing flexibility for free-flowing discussion where appropriate. Prompts were included to assist participants to focus on the issues relating to their own experiences. The issues discussed were designed to elicit information that would be useful for policy makers and health professionals planning to implement healthy lifestyle programs with adolescents. Facilitators had completed formal training with a qualitative research expert (AM) covering focus group conduct prior to involvement in these focus groups.

As per the focus group schedules, the stakeholder interview questions were developed then reviewed by an expert panel until consensus was reached. The stakeholder interviews were conducted by members of the research team. All interviews were recorded and transcribed verbatim.

### Analysis

With permission from participants, each focus group and interview was audio-recorded for accuracy of transcription and analysis. Confidentiality was ensured by not mentioning participant names whilst the audio-recorder was operating. Transcribed data were also de-identified with subject identifiers assigned to each participant. Data analysis was undertaken in stages, with focus groups and interviews dealt with separately. As soon as practicable following each focus group or interview (within 48 hours), responses to the questions were transcribed and initial thematic analysis conducted [37]. All focus group data was transcribed verbatim by one author (AM) and interviews transcribed by another author (KS). Content analyses of transcripts were completed by the authors responsible for the transcriptions to ensure consistency of coding. Inductive techniques were used to thematically code identified topics that emerged from the data [38]. The themes were then grouped into categories based around the structure of the three research questions. The themes and assigned categories were then validated by a second member of the research team and reviewed independently by the other authors to validate the themes thus adding to the overall credibility of findings and interpretations [39]. Differences in interpretation were resolved by consensus. The data were triangulated with adolescent, parent and stakeholder interpretations compared [40]. Summaries of the interviews were provided to stakeholders to allow member checking [41]. Any modifications were included in the analysis.

Data from focus groups and interviews were amalgamated and the major themes detailed in a report [42] using description and quotes from participants to support these findings [39].

## Results

Two focus groups were held with parents (n?=?4) and adolescents (n?=?4) who had participated in CAFAP, with written feedback provided by one adolescent and four additional parents who were not able to attend a scheduled focus group. All past participants who responded to the invitation had completed the full 8 week program. Four focus groups involving parents (n?=?4) and adolescents (n?=?13 per group) were conducted with potential participants. A total of 56 adolescents and adults provided feedback to the study, including13 past participants (n?=?8 parents, n?=?5 adolescents) and 43 potential participants (n?=?4 parents, n?=?39 adolescents). Adolescents were aged 12–16 years, with females comprising 52% of the sample. Of the parents, 82% were female. The majority of participants were white Australians from middle-low socio-economic areas. Details regarding household characteristics were not further explored. Focus groups typically lasted around 60 minutes.

A total of 26 interviews were conducted with 39 health professionals, local service providers and researcher stakeholders (see Table 1). All stakeholders approached agreed to participate and completed the interview, which typically lasted around 60 minutes. Interviews and focus groups were ceased when no new concepts or themes emerged and it was deemed that saturation had been reached.

**Table 1:** Background of stakeholder participants

**Interviewed**	**Profession**	**Background**
Health professionals	4 x dietitians	1 x private practice
		3 x country health
	4 x physiotherapists	4 x private practice
	2 x psychologists	1 x private practice
		1 x health department
	1 x general practitioner	1 x private practice
Local service providers	16 x state government	2 x health promotion coordinators
		1 x senior policy portfolio officer
		1 x community clinical nurse manager
		8 x community nurses (school health)
		2 x Parenting officers
		2 x sport and recreation representatives
	3 x local council employees	1 x youth services manager
		1 x youth Services Officer
		1 x leisure centre manager
Researchers	9 x researchers	From new South Wales, Victoria, South Australia and Western Australia

### Focus group and interview findings

Three major topics were discussed in the focus groups and interviews, to inform the design of more feasible and effective interventions. A summary of key findings are presented in Tables 2, 3 and 4 under these three topics being: 1) recruitment, 2) retention in the program and 3) maintenance of healthy change.

**Table 2:** Focus group and interview findings on perceptions regarding recruitment to a community-based healthy lifestyle program

**Recruitment**	
***We’ve had a lot of interest but it’s getting those families to actually register…and still wanting to attend (Allied Health Professional)***	
Barriers	Enablers
**Adolescents are often embarrassed about having to attend**	**Advertising needs to sell the message and promote it widely**
• *Teenagers often don’t want to go, because they’re very anxious they might see someone they know. Teenagers are already dealing with enormous bullying and other issues; to ask them to do something that they’re concerned may actually make their life worse is going to turn them off the project. (Researcher)*	• *It comes down to selling it really well and selling it as a healthy lifestyle thing, rather than a weight loss group. (Allied Health Professional)*
	**Message needs to be positive and not associated with being overweight**
	• *From a youth development perspective, it’s really important that the young people are interested in doing it, there’s a whole lot of stigma attached to identifying yourself as overweight or obese. (Local Council)*
**Overweight has become normalised**	
• *I think they’re in denial a lot of these parents…often the parents are overweight, the kids are overweight, the dog’s overweight, the cat’s overweight. (School Health Nurse)*	
	**Program needs to be free**
	• *The Government should see fit to subsidise something like this alright, ‘cause they keep talking about ‘we’ve gotta do something about the obesity of our children’. If they’re not going to put the money forward, then there’s… I mean I work two jobs just to try and make ends meet, I don’t sort of have the extra money to spend on stuff like this’. (Parent)*
**Reluctance to refer and lack of expertise in health professionals**	
• *Our experience is even paediatricians have had families come to them concerned but the family has been told ‘oh no they’re ok’ when they are clearly overweight, well into the overweight range. (Researcher)*	
**Lack of current services**	
• *The older people in the community are actually well catered for, but younger kids aren’t and I think seriously there is a huge gap because kids are just getting so overweight and they’re not fit. (Allied Health Professional)*	
**Broader social barriers**	
•* The only way you’re going to get them in is if it’s for free. The only way they’re going to keep coming back is if it’s for free. You’re not going to get a kid in a low socioeconomic family saying yep we’re going to put up the money for this kid [to access a program like CAFAP]. (Local Council)*	

**Table 3:** Focus group and interview findings on perceptions regarding retention in a community-based healthy lifestyle program

**Retention**	
***Most studies have real trouble getting the parents engaged and keeping them interested over time. (Researcher)***	
Barriers	Enablers
**Location**	**Good experience for adolescents**
• *I think it’s great if it can be more local, because I have broached it with some other parents before but either transport’s an issue or in trying to get off work and then get there after school, it’s a big ask. (School Health Nurse)*	• *I think anticipate that in any weight loss program, which is going to take months or years, people may well come in and out of it… If they see it is a good experience, if they see their teens happy, that’s probably something that’s going to really engage families. (Researcher)*
**Timing**	**Fun and practical**
• *Finding the time that actually works is very challenging. And it’s a barrier. (Researcher)*	• *It had to be fun, especially the adolescent sessions. It had to include fun, active games. They tended to bond more if you included those and when you look at the satisfaction questionnaires, they wanted more activity, as much activity as possible. (Researcher)*
**Commitment**	
• *The initial month or two is the hard part, because they’re going from nothing to exercising and always those first couple of months are hard. It’s hard for anyone. (Allied Health Professional)*	
	**Family involvement**
	• *Involving the family, is probably the most important thing that I see. Because it’s got to be a whole family change. Even if the particular teenager wants to do something, if the family’s not supporting that then it’s not going to go anywhere. (Allied Health Professional)*
**Social barriers**	
• *If you feel alone going there, that’s really bad. (Female Adolescent, Focus Group)*	
	**Use online components**
	• *Using electronic media too, that sort of validates it, if they’re getting reminders on their email or on their Facebook… even text messages. Maybe some online self-assessments- if they have something that they can go in and do their own little checklist and they get something back that says ‘oh you’re doing this now’ and prints some little graph for them about how they’re going. (Health Promotion Officer)*
	**Good facilitators**
	• *It’s really important about the people that you employ…as much as it’s about their proficiency and level of organisation, is how they interact, you almost need those social skills, they’re so important. (Researcher)*
	**Goal setting skills**
	• *One of the key aspects of goal setting is to make the goals realistic and achievable but also measureable. So that as they’re going along you can together assess whether in fact*
	• *Those goals are at any chance of being reached…because people want to be at the end. So if you can show them that they’ve had three steps forward and two steps back…but can still show them that they’ve made progress. That helps people stay engaged and have a sense of hope for change. (Psychologist)*
	**Easy and rewarding for parents**
	• *If the parent was coming along to that, the parent has got to get something out of it as well. That could be the exercise and all the same sort of things that you’re trying to do for the child. (Health Promotion Officer)*

**Table 4:** Focus group and interview findings on perceptions regarding maintenance in a community-based healthy lifestyle program

**Maintenance of healthy change**	
***Keeping them on track is really helpful, not just to go away and they forget all about it. (Researcher)***	
Barriers	Enablers
**Difficulty in sustaining change and keeping links to the program**	**Follow up**
	• *Following up with people…see how they’re going …keeps people a bit accountable and gives them a bit of motivation and reminders that we all need. (Allied Health Professional)*
• *The feedback from the kids and the parents is that they miss the regular contact and regular check in. I’ve had families specifically ring and say after a few weeks, ‘It’s not going well. I can’t do this and I need some support”. It’s like they need to set some goals and have someone else sit down with them and set some goals to keep going. (Researcher)*	
	**Positive changes are highlighted**
	• *If they see positive changes in themselves, whether it’s weight loss or they just feel better, I think if they see those changes, they’re more likely to carry that on…, they’re seeing benefits then that’s the biggest motivator”. (Allied Health Professional)*
**Lack of services to support change**	**Online/electronic media**
	• *I think text. All kids have phones, most parents have got phones. That’s what they hang off. (School Health Nurse)*
• *There’s a lack of centres or activities for kids who don’t want to be into sport, who may want to do something not as physical but with some physicality, but not in team sports. (School Health Manager)*	
	**Transition into community**
	• *Ways of linking them into community facilities as you kind of wean the program off. Looking at what’s available for them…So they’re exposed or it’s identified to them what opportunities are available in their environment so that there’s that potential for carry on. (Researcher)*

### Recruitment

Participants identified that recruitment of adolescents and families to a healthy lifestyle program was a critical issue. Participants recognised obesity as a current health problem and identified a need for interventions for overweight and obese adolescents, however, there were many potential barriers identified that may prevent adolescents from accessing these services. Participants suggested that the barriers need to be considered and addressed, where possible, to maximise the success of recruitment in the future.

[Recruitment was] very challenging. It took forever, took about twice as much time as we anticipated. And is the reason why we needed lots and lots of money. (Researcher)

### Barriers

Participants highlighted that adolescents can be a difficult group to recruit to healthy lifestyle programs for different reasons. For some adolescents, the fear of humiliation or bullying can make seeking help confronting, and for others, the promotion of a healthy lifestyle was not enticing if they weren’t overly concerned about their weight. In most cases, participants suggested that adolescent views regarding healthy lifestyle programs would be a barrier in itself.

I don’t think that adolescents would like to admit that they’re overweight. (Male Adolescent) Yeah. The reputation of having to go there [the program] and stuff. (Female Adolescent)

I don’t think that you should believe that young people will see those advertisements and say this is something I want to do. Even if it is something they want to do, they’re probably unlikely to say it. (Researcher)

It was identified that most parents don’t recognise if their adolescent is overweight, with overweight being almost normal in today’s society. This was thought to have the potential to reduce parent and adolescent receptivity to the offer of health services.

‘If your family think it’s ok to live like that, like nothing’s happened now, what would happen like three years later. And also if they’re already used to the fact that they’re obese, if they see someone suffering, say, going to the gym, and if their daughter or son’s getting stressed out from the exercise, they’ll think ‘oh, you’re ok being obese, let’s not do it’. (Adolescent)

Fundamentally in the general population, it’s not recognised as being a problem [that requires] something to be done about. (Researcher)

A hesitance to identify overweight and obesity by health professionals was raised. A number of health professionals identified the sensitive nature of obesity as a barrier to referral. A number of researchers identified other issues with health professionals not being able to measure children and adolescents to correctly identify overweight and obesity.

It’s a very sensitive issue. GPs said it is a really difficult thing to raise with parents if they haven’t raised it with you…They don’t want to jeopardise the relationship. (Researcher)

We wanted overweight and mildly obese young people…but we were being sent overly obese young people because these were the ones they saw…GPs don’t have a good way of assessing it…They don’t measure height and weight. GPs don’t know how to talk about it and paediatricians shy away from it. (Researcher)

The hesitancy of health professional referrals was reinforced by a lack of current community services. Researchers identified that new programs often struggled initially with attracting participants, particularly if there was no current referral base.

Up until now we haven’t really targeted obese kids because if we did, we had nowhere to go with it. OK we identify them but now what? (School Health Nurse)

I was looking for other things, particularly as she’s getting older and [dropping], tending to want to drop out of team sports and things like that or out of some of the programs at school that were keeping her very active. There’s nothing out there… Most gyms don’t even take them until they’re 15. (Parent)

Participants also identified that families often had a lot of social issues to contend with including; busy schedules, family problems, poor budgeting skills, a lack of healthy food preparation skills and other financial restrictions. It was expressed that often, healthy lifestyles were not a priority for these families.

It’s usually things are happening with social determinants or things are happening at home, yeah they’d like to eat healthy but Mum’s only got $20 for the rest of the fortnight and that kind of takes precedence. (School Health Nurse)

### Enablers

Participants recommended a wide-reaching and personalised advertising campaign to reach adolescents and parents. There was an emphasis on ‘selling the message’.

Face-to-face selling things goes a long way as well. It’s easy to put a brochure at the bottom of a school bag but if you actually talk to people and engage them…we can try and sell it. (Allied Health Professional)

Just generalised feedback about the whole group and what’s come out of it… If I see that someone I’ve referred has got something out of it, then [I’ll] definitely keep referring. (Allied Health Professional)

Promoting a positive message and trying to avoid embarrassing weight connotations were highlighted as important recruitment strategies.

[Do] anything you can to avoid the stigma of this being a project for overweight and obese. (Researcher)

If you promote it to help out their sport and improve their performance in that. Those sort of angles might be a good way. (Allied Health Professional)

Say ‘we’re about a lifestyle change’, not a diet, ‘cause that’s what you need to do, actually, a lifestyle change, otherwise you’re just gonna yo-yo for your whole life. Like feeling healthier, more than looking healthier. And feeling better within yourself’. (Parent)

Making a healthy lifestyle program available and accessible for all community members was an identified as an important enabler for recruiting adolescents and families. Participants recommended making the program free or very low cost to increase interest.

So you’re not forced to drop out for lack of money. (Adolescent)

Other parents suggested that making the program free would encourage attendance by families who weren’t totally committed to the program.

I think it was made free too, you might get people who might not really wanna be there for the right reasons, and it might be a bit too overcrowded. (Parent)

### Retention in the program

Participants described a need for healthy lifestyle programs to employ strategies to keep families engaged and interested, to help prevent drop out. Most researchers in particular had experienced the difficulties of keeping participants motivated to attend.

Following the initial sessions, attendance really dwindled, and sometimes yeah we had only one person. (Researcher)

### Barriers

The location and ease of access for participants was highlighted as important potential barriers for families to stay engaged with a program.

For many families that is a commitment, in our rather time poor community, that is quite difficult. And that’s why, presumably, success is partly due to having a site of study where it’s easy to get to. (Researcher)

Another program-specific factor of start and finish times was identified as a barrier that may make it difficult for some families to stay engaged. Participants were conflicted in their view for the most appropriate start time, wanting to include adolescents immediately after school, but noting that parents are often not available at this time with work and family commitments.

So many parents, if not full time, are working part time… People struggle to pick up their kids from school and get there. (Researcher)

Stakeholders were quick to acknowledge that attending an ongoing healthy lifestyle program and making healthy lifestyle changes were difficult things to do. They noted that the program needed to be a priority for the family and facilitators would have to work hard to try and keep families motivated.

Bigger the body mass, the bigger the resistance to change- partly through a sense of being overwhelmed. Like how am I ever going to be a size 10 if I’m a size 24. If I can’t be a size 10 then I’m not going to bother. (Allied Health Professional)

This is difficult and emphasising that this hasn’t happened overnight and it isn’t going to go away overnight. You need to commit as a family and so we emphasise that family thing. (Researcher)

Participants identified that the environment we live in makes it difficult to stay engaged and make healthy changes.

McDonalds has come out with an ad for under $5 they can get a burger and this and that and the other. You’ve got the convenience and low cost of high salt, high fat junk food. How do you get healthy food choices that are cost effective, easy to prepare and that they’re interested in, when there’s all the attractiveness of this junk food. (Health Promotion Officer)

### Enablers

Stakeholders recommended focussing on making the program enjoyable and rewarding for adolescents to increase the chance that the family would remain in the program.

Would be great to train with someone else in the group. Random assignment would mean you meet more people. [You] could ‘tag-team’ one exercise until you can’t go anymore. (Adolescent)

Just a group type session, particularly teenagers- they’re one of those groups, and if you get together and they’ve all got similar problems then it’s a lot easier for them to work through those problems and come up with solutions… It’s really hard when they’re on their own, if they feel like they’re on their own. (Allied Health Professional)

There were suggestions for the program content, such as using activities that are fun and active, as well as providing practical skills like cooking.

I think once they get engaged and see that it’s practical, then they’ll be fine…and when it gets a name for itself and they can see changes in other teenagers. (School Health Nurse)

It was really very easy to knock up snacks and do stuff that was appropriate for teenagers. And I still maintain that you can eat healthily at a reasonable price, I like [this kind of program] as adapted to a teenage market, not for a mum and a couple of kids. (School Health Nurse)

The importance of including the whole family was highlighted by all focus group participants, even by the adolescents themselves.

Cause it’s also a lot about the parents. You need to get the parent involved because, like you said, they’re in control of the food and, like, the computer playing and stuff. So basically you have to talk to the parent I guess, and then make them see what they’re doing to their child- they have to do this. (Adolescent)

Participants described program staff as one of the key enablers for keeping families engaged in a program. Passionate, interested and motivated facilitators were seen to increase the engagement of parents and adolescents in the program. Researchers described the development of a good relationship between facilitator and participant as one of the most critical aspects of the program.

I think the only thing that would really stop somebody would be a huge personality conflict, right, with the kids with the trainers, instructors, whoever is running it, ‘cause if the child doesn’t like the person, they’re not gonna sit there and listen’. (Parent)

Certainly how well a group runs and how well it all goes does depend on the facilitator and the relationship they build. (Researcher)

The use of goal setting during programs was discussed as a good way for adolescents and parents to make small changes and see the progress they make.

I think goal setting is really important because people can get confused and they can get overloaded. And so it’s the sort of standard suggestions that are made in CBT and other things, you pick a goal that’s achievable. You pick a goal that somebody will understand. You look at pathways to achieve that goal. It is important to let teens personalise things… it should be simple and attempting the goal is praised in some way. (Researcher)

To account for the effort required to stay engaged in a program, participants recommended making it as easy as possible for families to attend and rewarding their attendance with incentives or teaching them new and practical skills.

With disadvantaged families in particular, those kind of altruistic ‘your life’s going to wonderful if you do this’, isn’t going to get them there. You’ve got to have practical things like we’re going to give you a gift card or you’re going to get a shopping voucher…That’s actually the kind of thing you’re going to need with disadvantaged parents. (Health Promotion Officer)

### Maintenance of healthy changes

Participants unanimously agreed that maintaining healthy behaviour changes after being involved in a healthy lifestyle program was difficult and required a lot of motivation and commitment from families. It was acknowledged that for adolescents, ways to encourage sustainable change is lacking good evidence and there is still plenty of work to be done in identifying enablers in this maintenance period.

We know little about, except for some work from the States from the obesity register of why adults keep the weight off, we really don’t know what happens in adolescents. (Researcher)

### Barriers

The difficulty in sustaining healthy changes, especially in the context of other family issues was noted by stakeholders. Researchers in particular identified that it was difficult to organise convenient times or interesting activities to encourage adolescents to come back to review or booster sessions to keep up the support from the program.

Some families go great guns, you know they’ll keep going with things. I guess that’s when there’s no conflict, no social issues and the kid’s really motivated…but there’s some families that you probably know, because of the kid or the parent or both, they’re going to fall in a heap. (Psychologist)

One thing is, as the adolescents got older they ended up getting part-time jobs, or they had greater study commitments, so I think that’s one reason why attendance tapered off in the booster sessions. (Researcher)

Participants identified a lack of supportive services in the community to encourage overweight adolescents to maintain healthy changes. They noted that these adolescents were unlikely to re-engage in team sports but there are few activities available in the community for them to access instead.

We struggle when families get to the end of those twelve months and they want more support, there’s not really anything to refer them onto. (Researcher)

My daughter’s doing Year 8, she’s doing home economics. Guess what they’re cooking? Chocolate cake, simple as that. I mean, it’s nice to have, but they’re not taught that it’s nice to have a little bit, yes, and once in a blue moon it’s ok; but they don’t, they sit there and they have it for morning tea, chocolate cake. (Parent)

### Enablers

A number of factors were suggested by participants that could facilitate maintenance of healthy lifestyle changes. An ongoing link to facilitators or program staff was identified as a potential enabler after the program has finished.

We use things like postcards at Christmas time…maybe here’s some things to think about at Christmas. Trying to get that connection. (Researcher)

An email or check in point where a couple of months down the track…they send a coordinator a message saying these were my goals and this is what I’m doing. Just to sort of make them still take ownership of those goals that they’ve set. (Allied Health Professional)

Participants highlighted the importance of adolescents and parents feeling like they were capable of achieving their own healthy change and having these positive changes recognised by themselves and others when they occurred.

They’ve got to have buy in. And it’s absolutely essential that the parents are involved in it if you want to change things. And you want them to have seen changes…and believe they can do it. (Researcher)

Communicating with adolescents using their preferred means of contact, particularly by SMS and online communication, was highlighted as a good way to encourage maintenance of healthy change.

IT- It’s a cheap, simple and effective way of maintaining engagement. (Psychologist)

I think we really need to explore all of those forms of e-communication that young people do, and just use them as much as we can because they’re forever SMS-ing and Facebooking and so on. And we just need to be using that as part of our ongoing ways of connecting to them. And the dose we were thinking is just way too small. (Researcher)

Participants agreed that transitioning adolescents into local services and groups after the programs was an important part of maintenance. They were able to identify some local services that may be accessed to provide opportunities for encouraging kids to stay active (e.g. sporting clubs) and mentally well (e.g. youth services) but highlighted the need for alternative options for adolescents who didn’t enjoy sport.

I think the kids at 12–16 that aren’t involved in sport, I’d dare to say they’re probably not going to be interested in sport in the future. So you probably need to think maybe like the nature play type activities, the trail bike riding or the bushwalking or canoeing or those sort of sports. (Local Council).

Some kids just don’t like sport… It’s trying to educate them on what they enjoy doing. Sometimes you might do things at home so they can set up a little system at home or an aerobics video- lots of videos and things out there now. (Allied Health Professional)

## Discussion

Past CAFAP participants, future potential CAFAP participants and community stakeholders involved in supporting health interventions articulated a rich description of possible barriers and enablers to recruit and retain future adolescent healthy lifestyle program participants. They also discussed possible enablers for overweight adolescents to maintain a healthy lifestyle after the completion of a program like CAFAP. Some ideas were consistent across informants and supported by existing literature, whilst other emergent ideas and opinions differed between groups and participants.

A strong theme emerged regarding the need for a positive awareness-raising campaign to encourage recruitment. The stigma associated with being overweight or obese was identified as a major barrier by all participant groups, and potentially prevented adolescents looking for help. This is consistent with the literature suggesting overweight adolescents are at greater risk of social isolation and depression [6,7]. This also highlights a need to steer away from labelling adolescents as overweight or obese and labelling programs as weight-related interventions; toward healthy lifestyle or skill-based programs. There were a number of other strategies to improve recruitment highlighted by all groups interviewed in line with current literature, including the importance of advertising the program widely [21,33], particularly on the internet, and the need for strong links with health professionals [33], particularly General Practitioners (GPs), to inform them of the program and ways to discuss it with parents and adolescents. Stakeholders indicated that they felt GPs and other health professionals were hesitant to raise the subject with parents or adolescents, most often due to a lack of referral options or lack of training to effectively manage these patients. This is consistent with existing literature [43,44].

An important theme relating to keeping adolescents and parents engaged in the program was to make it accessible and enjoyable for participants. The importance of a local venue and a convenient time was emphasised by all groups interviewed, supported by Brennan et al. [24] who found that non-completers of an adolescent weight management program were more likely to drop out if: there were travel or transportation difficulties; it took too long to get to the sessions; or if work schedules interfered with the timing. A need for passionate and engaging facilitators was raised, with recent literature suggesting that community organisations should recruit appropriately skilled program leaders to maximise retention [33]. Participants agreed that the program needed to be fun for all attendees, with a focus on practical activities to keep adolescents and parents engaged; a concept well supported in the literature [45,46]. Whilst not specifically asked, participants did not question the desirability of having both parents and adolescents involved, in line with current recommendations [8,47].

All groups raised the issue of difficulties committing to healthy lifestyle changes and suggested that adolescent ‘buy in’ was critical. Maintenance of healthy lifestyle behaviours are often associated with motivation fostered during the process of attaining goal-related behaviours [48], and requires one to be committed to the goal for behaviour change to ensue [49]. Previous qualitative work with obese adolescents suggests that they become less motivated within a short period of time [50] and that emphasis needs to be placed on addressing underlying factors for excess weight gain and setting realistic goals for change. Adolescents’ autonomous motivation, or their sense of volition and enjoyment regarding behaviour engagement, has consistently predicted sustained engagement in healthy lifestyle behaviours [51] and capitalising on this may help to keep adolescents engaged in healthy behaviours. This idea has been incorporated into the goal setting structure to use with obese adolescent in the refinement of our healthy lifestyle program [52].

A strong theme emerged regarding the lack of local, accessible, affordable and enjoyable physical activity options for adolescents in the community to support maintenance of healthy behaviour change following a healthy lifestyle program. This presents a significant barrier for adolescents who may have the skills and motivation to change but are restricted in their environment. This fits with the proposed Ecological Systems Theory Model of predictors of childhood overweight [36], where low availability of recreation facilities and safe places to be active have a negative impact on children’s activity levels, and hence their health and weight [36]. More research is needed to understand preferred physical activity options for overweight adolescents, with adolescent studies suggesting non-formal exercise like cycling or walking, which does not require a high level of skill [50].

Opinions differed regarding the cost for families to attend a healthy lifestyle program, with some parents and stakeholders suggesting a small monetary fee would encourage more ‘dedicated’ attendees and help people to place more value on the program. Other stakeholders and parents indicated that parents would be put off attending if there was a cost associated with the program and these differing opinions are highlighted in two reports of stakeholder views [33,53].

Common barriers for parents of adolescents who dropped out of a recent lifestyle intervention included a lack of interest/motivation and lack of time available to participate [24]. Parents involved in the focus groups described high levels of motivation which may not be reflective of the opinion or intent of all parents of overweight adolescents and this research should to be interpreted in the light of participant bias [54]. Previous research with parents of obese adolescents suggests high levels of concern for their adolescent’s well-being but often a sense of helplessness with how to work with their adolescent [55]. This study confirms parent concern but further insights are limited by small group numbers.

There were a number of limitations with this study. Firstly, difficulties with recruitment resulted in fewer adolescent and parent participants than planned. However, an absence of new topics emerging from the final transcriptions suggested saturation had been reached for parents and adolescents as well as stakeholders. This research explored the opinions of adolescents, parents and stakeholders in low-middle socio-economic regions of Western Australia and thus results need to be taken in context. We had some minority ethnic groups represented but no indigenous participants in the focus groups, so findings cannot necessarily be generalised to different communities. The views of researchers were open to bias as they were reporting on their experiences with overweight adolescent interventions. However, researchers were only included in the interviews if they had extensive and direct experience in this field, which the authors feel is of high clinical significance. Further, lifestyle interventions may only reach the sub-group of obese adolescents willing and able to attend such programs and thus other interventions such as active desks [56] are needed to reach the whole population in need. Despite these limitations, the results from this study are consistent with the current literature regarding recruitment and retention for community-based programs [57].

The strengths of this study include consultation with, and direct input from adolescents, their parents and key stakeholders around the prevention and/or management of adolescent overweight and obesity. Trained facilitators led the discussions and the homogenous groupings permitted honest and open discussion in a supportive environment. The discussion schedules were based on a sound theoretical framework with questions tailored to explore individual, familial and broader societal factors associated with adolescent obesity. Other strengths of this study were consultation with a wide range of informants with relevant experience in the area, and the collection of in-depth information about the challenges associated with the implementation and evaluation of interventions for overweight adolescents by examining the whole spectrum of recruitment, retention and maintenance.

Despite accumulating research into perceptions of adolescents, parents and stakeholders regarding the causes of overweight and suggested management strategies; there has been limited evidence to inform the actual implementation of a community-based healthy lifestyle program. This study makes unique a contribution to the evidence base by describing the barriers and enablers to implementing a successful healthy lifestyle program for overweight adolescents as perceived by families and community stakeholders. This understanding will be useful to improve acceptance, attendance and completion of programs thus improve their feasibility within a community setting.

### Implications for research and practice

Based on the valuable information provided by past participants, potential participants and stakeholders, a number of recommendations are made to maximise the effectiveness of a family-centred, community-based intervention for overweight adolescents. These recommendations have already guided the protocol for one such program [34].

Recruitment is one of the most difficult parts of any lifestyle program. Researchers and health professionals need to utilise a number of strategic marketing approaches to attract parent and adolescent interest including: online advertising and web-based information; advertising in newspapers and on radio; repeated exposure; and development of links with schools and health professionals [33]. Potential barriers to engaging in a healthy lifestyle program also need to be identified, considered and minimised within each community where possible, particularly regarding location, timing (both days of the week and start/finish times) and cost.

To retain families and keep them engaged with a healthy lifestyle program, facilitators need to be passionate and engaged themselves, with time and focus given to fostering relationships with both adolescents and parents. This sense of belonging and opportunity to share similar problems plays an important role in a successful group. Adolescents also need to have opportunities to make choices about what they do and provide feedback about the program. The sessions need to be fun, with a positive focus on practical activities. Goal setting based on these factors also plays an important role to help participants work towards realistic and achievable goals, whilst having the potential to demonstrate progress and promote autonomous motivation. The activity component of the sessions should be fun, with a number of activities that adolescents may not have done before and may be interested in continuing with once the program has finished. Ultimately, parents and adolescents need to feel that the program meets their needs, with content relevant and useful for the families who attend.

There has been little research into maintenance of healthy behaviours after adolescent participation in a healthy lifestyle program. This study found that to maintain ongoing positive change, follow up contact needs to be regular and appropriate to assist with goals set during the program. Participants should stay linked to the program after it has concluded and programs may need to explore online support as a strategy to do this. Setting up a website with recipes, tips for exercise, goal-setting activities, and testimonials from former participants could keep families engaged with the healthy lifestyle changes and an ability to interact with other participants, for example through a blog or social network site, would provide networking opportunities for both parents and adolescents. Special attention should be given to developing multi-media strategies to suit adolescents. Maintenance of healthy changes after program conclusion could be supported further by linking adolescents with existing community services that promote being active, eating healthily or engaging in community activities. This should be promoted during the program and adolescents could choose activities or programs they enjoy. Overweight adolescents may be reluctant to re-engage in organised sport, so other options like cycling or fitness classes should be encouraged.

Assessment burden needs to be minimised or compensated for where possible. It should be recognised that lengthy or invasive assessments have the potential to dissuade participants from staying engaged in an intervention program. Assessments need to be kept as short as possible, only measure the specific outcomes associated with the program and be completed at a local site that can be easily accessed by participants. Compensation or incentives for completing assessments may need to be provided if the burden cannot be minimised sufficiently*.*

## Conclusions

Being overweight in adolescence is a major problem; however, limited evidence is available regarding effective and appealing intervention programs for overweight adolescents. Previous studies note difficulties with recruitment or high drop-out rates, however, there is limited evidence identifying specific barriers and enablers to engaging overweight adolescents in a healthy lifestyle program, how to keep them engaged and how to maintain healthy behaviour changes post intervention. This study outlines a number of key barriers to recruiting adolescents and families, and suggests ways to maintain engagement and behaviour change during and following a healthy lifestyle program for overweight adolescents and their families. These findings can be used by researchers to enhance recruitment, retention and maintenance in community-based intervention studies with the target group. The findings can also be used by health service policy makers, planners and service providers to improve feasibility and acceptability of these types of programs and their long term institutionalisation within community settings.

## Competing interests

The authors declare that they have no competing interests.

## Authors’ contributions

KS conducted focus groups and the interviews, analysed the data and drafted the manuscript. LS conceived of the study, and participated in its design and coordination and helped to review the manuscript. AM conducted focus groups, transcribed data, assessed themes and helped to review the manuscript. AAF contributed to the interpretation of the results and helped to draft the manuscript. All authors read and approved the final manuscript.

## Pre-publication history

The pre-publication history for this paper can be accessed here:

http://www.biomedcentral.com/1471-2431/14/53/prepub

## Supplementary Material

Additional file 1 **Parent and adolescent focus group prompts.** Discussion points for past and potential participants. Click here for file

Additional file 2 **Stakeholder interview prompts.** Discussion points for health professionals, local service providers and researchers. Click here for file
