# Supplementary material for: Barriers and enablers for participation in healthy lifestyle programs by adolescents who are overweight: a qualitative study of the opinions of adolescents, their parents and community stakeholders
Source: BMC Pediatr. 2014 Feb 19;14:53. doi: 10.1186/1471-2431-14-53 (PMC3942615; doi:10.1186/1471-2431-14-53)
Supplement: Additional file 1 — Parent and adolescent focus group prompts. Discussion points for past and potential participants. [file 1471-2431-14-53-S1.docx]

**Past participants (parents and adolescents) - Focus group discussion prompts**

*Black dot points are the main prompts, white dot points are follow up prompts.*

Finding out about and getting interested in the program

- What was it that made you enquire about CAFAP in the first place?
  - Was there anything that almost stopped you from enquiring?

Staying with the program

- What made it difficult to stay engaged in the program?
  - When filling out the forms, it was sometimes difficult for parents and kids to do these, or remember to bring them back. How can we make this process easier?
  - Would assessments be better done in the home or at the clinic/Curtin?
  - We have noticed that some families have had issues with:
    - Distance/transport
    - Time
    - Length of program
    - Other after school commitments
    - Lack of interest
  - How can we deal with these?
  - ADOLESCENT SPECIFIC
    - How can we keep the motivation up in the gym? Would a buddy system work?
    - Was it important for you to have people in the group who were the same age/gender as you?
- What were the benefits of staying with the program?
- When completing ‘at-home’ activities, it was sometimes difficult for parents and kids to remember to do these. How could we make this easier?
  - Would you like a reminder? (SMS, phone, email, electronic media?)

Keeping up with the changes

- What did you change as a result of the last program? Why?
  - What stopped you from making other changes?
  - Is there too much or not enough information in the program? Would we better to focus on a couple of main ideas and refresh this several times?
  - How well do you think the professionals from physiotherapy, dietetics, psychology and social work actually worked together to make the program fit together and make sense?
  - Do you have any suggestions for how the key messages or activities delivered by the different professionals (physiotherapist, dietician, psychologist and social worker) could have fitted together better?
  - Do you refer to the goals you set during the program? If not, how can we make goal setting more useful for your family?
- Sometimes it can be difficult to get families back for testing at 3 and 6 months. How can we make this easier, or how can we provide incentive?
- What kind of ongoing support would have been helpful after you had completed CAFAP?
  - Would you like to be contacted by SMS, phone, email, electronic media?
  - Are there any services in your community that we can help you get/stay involved in?

**Potential participant focus group discussion prompts**

*Black dot points are the main prompts, white dot points are follow up prompts.*

**Parents:**

Finding out about and getting interested in the program

- What would get you interested in a program like CAFAP?
  - Who would you ask or where would you look for information about a program like CAFAP?
  - Here is a sample of our advertising flyer. What interests you? How would you immediately know that this program may be good for you and your family?
  - What would be the most important things for CAFAP to do to help adolescents take some interest/be willing to find out more about the program?
- What would prevent you from being involved in a program like CAFAP?
  - What would put you off enquiring about a program like CAFAP?
  - What would put you off being involved in CAFAP?
- Why would you like to be involved in a program like CAFAP?
  - What are the key concerns you have about your child’s health/weight?
  - What behaviours concern you? *(may indicate ways to target parents’ main concerns)*
  - What would you expect to get from a program like this?
  - CAFAP is not a weight loss program. Our program focuses on healthy lifestyle changes rather than weight loss. With a healthy diet, regular exercise and healthy attitudes, we find that body weight will respond and stabilise on its own accord. If this is different to your expectations, how could we explain this best or sell this idea to you?
  - From the flyer, what do you understand to be the role of parents in the CAFAP program? Our program includes parents in all sessions and their active participation is integral to creating healthier lifestyles for adolescents. How can we ensure that from the beginning, parents have a clear understanding about their important role in the program?

Staying with the program

- What are the general barriers to being and staying involved in a program like CAFAP? How could we overcome these?
  - We have noticed that some families have had issues with:
    - Distance/transport
    - Time
    - Length of program
    - Other after school commitments
    - Lack of interest from child
  - How can we deal with these?
  - How close to your home/school would the program need to be?
  - This program includes parents in every session (2 hours, twice a week for 8 weeks). How can we encourage parent involvement/ make this more inviting for parents?
  - This program includes an hour of testing/data collection before the program, after the program and at 3 months after the program. Would assessments be better done in the home or at the clinic/Curtin?
  - We ask adolescents and parents to complete a number of forms before and after the program to provide information about any changes in their behaviours and feelings, to help us evaluate the effectiveness of the program. Sometimes parents and kids find it difficult to do these, or remember to bring them back. How can we make this process easier?
- What would you identify as positive aspects of this program that may interest other parents? *(may indicate ways to target parents’ main motivators)*

Keeping up with the changes

- What kind of ongoing support do you think would be helpful once your family had completed a program like CAFAP?
  - Would you like to be contacted by SMS, phone, email, electronic media?
  - Are there any services in your community that we can help you get/stay involved in?

**Adolescents:**

Finding out about and getting interested in the program

- What would get you interested in a program like CAFAP?
  - Who would you ask or where would you look for information about a program like CAFAP?
  - Here is a sample of our advertising flyer. What interests you? How would you immediately know that this program may be good for you?
- What would put you off being involved in a program like CAFAP?
  - What would put you off telling your family about a program like CAFAP?
- Why would you like to be involved in a program like CAFAP?
  - What would you expect to get from a program like this?
  - CAFAP is not a weight loss program. Our program focuses on healthy lifestyle changes rather than weight loss. With a healthy diet, regular exercise and healthy attitudes, we find that body weight will respond and stabilise on its own accord. If this is different to your expectations, how could we explain this best or sell this idea to you?

Staying with the program

- What are the general barriers to being involved in a program like CAFAP? How could we overcome these?
  - We have noticed that some adolescents have had issues with:
    - Not knowing other teenagers in the group
    - Time (after school)
    - Length of program
    - Other after school commitments
    - Lack of interest

How can we deal with these to encourage you to join in?

- - Would you need to know if there are other participants in the group who are the same age and/or gender as you?
  - We need to have a number of forms filled in before and after the program. Sometimes parents and kids find it difficult to do these, or remember to bring them back. How can we make this process easier?
- What would you identify as positive aspects of this program? *(may indicate ways to target adolescents’ main motivators)*

Keeping up with the changes

- What kind of ongoing support do you think would be helpful once your family had completed a program like CAFAP?
  - Would you like to be contacted by SMS, phone, email, electronic media?
  - Are there any services in your community that we can help you get/stay involved in?
