# Supplementary material for: Barriers and enablers for participation in healthy lifestyle programs by adolescents who are overweight: a qualitative study of the opinions of adolescents, their parents and community stakeholders
Source: BMC Pediatr. 2014 Feb 19;14:53. doi: 10.1186/1471-2431-14-53 (PMC3942615; doi:10.1186/1471-2431-14-53)
Supplement: Additional file 2 — Stakeholder interview prompts. Discussion points for health professionals, local service providers and researchers. [file 1471-2431-14-53-S2.docx]

**Stakeholders: Interview prompts**

**Health professionals (including community health nurses, GPs, allied health workers)**

Practice and management

- Do you currently see overweight/obese teenagers in your practice?
- How many or how regularly would you see overweight/obese teenagers?
  - For what proportion would overweight/obesity be the primary presenting complaint?
  - Would you consider mentioning the teen’s weight if it wasn’t their primary complaint?
  - For those teenagers you do see for overweight/obesity, how do you manage them?
- What guides your service delivery?

Community services

- What are the strengths of services offered?
- What are the current gaps in services for overweight teenagers and their families?
- Ideally, what kind of services would you like to be available to overweight/obese teens?
- What facilities are around that may be used to deliver a lifestyle program to overweight teens? (CAFAP needs a meeting room, exercise equipment area and basic food preparation facilities)
- What are the costs associated with using such facilities?
- Are you involved in or aware of health-related groups that run successfully in the community (not necessarily for adolescents)? What can we learn from them?

Recruitment

- What do you think are the main issues in getting people interested and enrolled in a program?
  - What works for you?
  - How did you try to overcome any barriers?
  - If you knew that CAFAP existed, what kind of information would you want to know about it?
  - Here is a sample of our advertising flyer. How would you immediately know that this program may be good for families you see? What other information would you need?
  - Who is well-placed to refer or recruit teenagers?
  - How do we best get our referral information to you? Or other referrers?
  - How would you mention this program to families? Would you feel comfortable talking about this?
  - What could we provide (for health professionals or families) to make this discussion easier?
  - What level of feedback would you like about participants you have referred?

Retention

- What do you think are the main issues in keeping individuals/ families engaged in health services?
  - Did you have any difficulties keeping individuals/families involved in your practice?
    - Why do people dis-engage?
    - How did you try to overcome these barriers?
  - Do you have any suggestions about using goal setting to help keep individuals/families engaged?
  - What do you think individuals /families respond well to that keeps them engaged?

Maintenance

- What do you think are the main issues in helping individuals/ families maintain positive healthy lifestyle changes after a program?
- What support services can we link participants with once they have completed the program?
  - How do you know about these services or how do others find out about them?
- Have you got any ideas or comments about sustaining positive healthy lifestyle changes?
- Are there other people that may have valuable insight into this area that you think we should speak to?

Program

- If CAFAP was to be implemented in your local area, is this something that you or your organisation would support? What support would you be able to provide?
- Would you be interested in being involved in the implementation of this program in your local community?
  - What interests you and what puts you off?
  - What else should our team consider?

**Researchers**

- Could you briefly outline your experience researching issues related to obesity intervention programs for adolescents
  - Intervention studies – age group, nature of program
  - Other research – reviews, assessment method development…
- What do you think are the main issues in getting families interested and enrolled in a program?
  - Did you have difficulties recruiting overweight/obese teenagers?
    - How did you try to overcome these?
  - We have recruited families through advertisements in the Parent’s Paper and community newspaper, local radio ads and inclusion of information in school newsletters. We have also written to GPs and Allied Health professionals in the area and asked them to refer appropriate clients. How can we improve our strategy?
  - We aim to target overweight and moderately obese adolescents (have previously mainly recruited severely obese). What different issues may this raise?
- What do you think are the main issues in keeping families engaging in a program?
  - Did you have any difficulties keeping families involved in your program?
    - How did you try to overcome these?
  - This is a timetable summary of our current program. I can explain/ give you some more detail about program content if needed. Do you have any suggestions for improvement t help engagement?
  - This is a list of the assessments we have previously included before and after the program. Do you have any suggestions for reducing the burden and improving the utility?
  - Do you have any suggestions about using goal setting to help keep families engaged?
- What do you think are the main issues in helping families maintain positive healthy lifestyle changes after a program?
  - How have you helped overweight teens to maintain their healthy changes once they finished your program?
  - We are considering using IT (SMS/Email/Facebook) to encourage maintenance. Do you have any comments or ideas about how to do this well?
  - Do you have any suggestions about integrating participants into community run physical activity?
  - Any other suggestions to help maintain lifestyle changes?

**Council:**

- What services are currently available for overweight teenagers in this local area?
- What are the strengths of services offered?
- What are the limitations of services offered?
- Who is involved in offering services?
- Who is well-placed to refer or recruit teenagers?
- What facilities are around that may be used to deliver a lifestyle program to overweight teens? (CAFAP needs a meeting room, exercise equipment area and basic food preparation facilities)
- What are the costs associated with using such facilities?
- If CAFAP was to be implemented in your local area, is this something that your organisation would support? What support would you be able to provide?
- What support services can we link participants with once they have completed the program?
  - How do you know about these services or how do others find out about them?
- Are there other people that may have valuable insight into this area that you think we should speak to?

**Policy makers:**

- Do you know of any current services available for overweight/obese teenagers?
  - Who organises these services?
- Ideally, what kind of services do you think needs to be available for overweight/obese teens?
- We hope to implement CAFAP in two metropolitan communities later this year. Is there anything that we need to know about the way health services are currently delivered? (eg/ clinical pathways, service priorities, health professional limitations)
- Would your organisation/service have any capacity to be involved in:
  - Recruitment/referral of overweight teenagers
  - Delivery of CAFAP
  - Maintenance programs/support
- Would your organisation be happy to endorse or support the implementation of CAFAP?
- How can we best work with you in the future?
